# Association of personal and equipment-related factors on ACL injury risk in alpine skiers with cautious or risk-taking behaviour: A case-control study

**DOI:** 10.3934/publichealth.2023026

**Published:** 2023-05-06

**Authors:** Gerhard Ruedl, Markus Posch, Elena Pocecco, Katja Tecklenburg, Birgit Schliernzauer, Michael D. Kennedy, Martin Faulhaber, Martin Burtscher

**Affiliations:** 1 Department of Sport Science, University of Innsbruck, Innsbruck, Austria; 2 Medalp sportclinic, Imst, Austria; 3 Faculty of Kinesiology, Sport and Recreation, University of Alberta, Van Vliet Ctr, Edmonton, Alberta, T6G 2H9, Canada

**Keywords:** risk-taking behaviour, alpine skiing, ACL injury, ski geometry, standing heights

## Abstract

**Background:**

In recreational alpine skiing ACL injury risk depends on the interaction of individual characteristics and behaviours as well as on equipment-related factors.

**Aim:**

to evaluate if and to what extent personal characteristics and equipment-related parameters are associated with ACL injury risk in cautious and risk-taking recreational alpine skiers.

**Methods:**

A retrospective questionnaire-based, case-control study of ACL-injured and uninjured in a cohort of cautious as well as risk-taking recreational skiers was conducted. Participants self-reported their demographics, skiing skill level, and risk-taking behaviour. Ski length, side-cut radius, widths of the tip, waist, and tail were recorded from each participant's skis. Standing heights at the front and rear components of the ski binding were measured with a digital sliding caliper, and a standing height ratio between the front and rear components was calculated. Ski boot sole abrasion at the toe and heel pieces was also measured with the digital sliding caliper.

**Results:**

In total, 1068 recreational skiers (50.8% females) with a mean age of 37.8 ± 12.3 years participated, of whom 193 (22.0%) sustained an ACL injury, and 330 (30.9%) participants reported a risk-taking behaviour. Results of the multiple logistic regression analyses revealed that a higher age, a lower skill level, a higher standing height ratio, and greater ski boot sole abrasion at the toe as well as heel pieces were independently associated with an increased ACL injury risk in both the cautious and the risk-taking group. Among cautious skiers, a longer ski length was an additional significant risk factor for sustaining an ACL injury. In conclusion, the same personal and equipment related characteristics contribute to an increase in the ACL injury risk regardless of risk-taking behaviour, with the only difference that longer skis represent an additional risk factor in cautious skiers.

## 1. Introduction

Reported cases of anterior cruciate ligament (ACL) injury is a growing cause of concern in sport and recreation including recreational alpine skiing. In fact, ACL injury is the most common diagnosis in recreational alpine skiing accounting for 20% of all ski injuries [1],[2]. Furthermore, in about half of all knee injuries the ACL is affected [3],[4]. Skiing-related ACL injuries are usually the consequence of an inciting event, i.e., a fall or a collision, which may result from a complex interaction between internal and external risk factors [5]. While potential external factors for a skiing-related injury include the equipment used and environmental factors such as snow quality, potential internal factors such as sex, age, body composition, fitness, and skill level have also been identified [5]. Interestingly, psychological factors such as perception of risk are also causative of ski injury [5]; however, the influence of risk-taking ski behaviours has not been fully explored, especially in musculoskeletal injuries such as those at the knees.

In past years, the association between risk-taking behaviour and ski helmet use according to the so-called risk compensation theory has been studied extensively [6]–[11]. For instance, Ruedl et al. [12] showed that self-reported risk-taking behaviour on ski slopes was associated with younger age, higher skiing ability, male sex, a lower body mass index (BMI), and higher measured speeds, but not with helmet use. In another study on uninjured skiers and snowboarders, male sex, younger age, skiing (vs. snowboarding), a higher skill level, a higher mean skiing time per season, and higher scores on the sensation seeking scale, but not ski helmet, were predictive of self-reported risk-taking behaviour on ski slopes [13]. In contrast, other studies have shown that wearing a helmet increases self-reported risk-taking behaviours [9]–[11]. Although it is an on-going discussion whether wearing a ski helmet increases individual risk-taking behaviour, there are evidence-based arguments that risk compensation does not undo the protective effect of ski helmet use [14]. Nevertheless, the question still remains whether equipment used affects individual risk-taking behaviour or whether individual risk-taking behaviour affects the choice of equipment used.

Other studies have evaluated the impact of individual risk-taking behaviour on injuries while skiing [15],[16]. Bouter et al. [15] found no difference in the general sensation seeking scale between injured and uninjured skiers, but the Thrill and Adventure Seeking (TAS) subscale was significantly higher in the uninjured skiers. These authors concluded that skiers with a high TAS score are better at handling the risk of several forms of physical exercise and thus less prone to accidents and injuries [15]. Interestingly, Goulet et al. [16] have found that injured skiers did not take more risk but were less skilled compared to uninjured skiers while those skiers who were identified as risk takers on ski slopes were the most skilled [16]. This research illustrates that skill is an important factor in risk-taking behaviour as shown in other research which found that self-reported risk-taking behaviour on ski slopes was associated with a higher skill level [17]. However, Ruedl et al. [17] also found in a cohort of more than 2000 injured skiers and snowboarders that the cause of the accident did not differ significantly between risk-taking and cautious people.

On the other hand, Niedermeier et al. [18] reported that winter sport participants with treated injuries showed significantly higher sensation seeking compared to uninjured people. Also, Ruedl et al. [19] found self-reported risk-taking behaviour on ski slopes as an independent risk factor for suffering an ACL injury. Thus, the preceding evidence on how risk behaviour and thrill-seeking sensations is predictive of ski related injuries including knee injury is equivocal.

There is evidence, however, that self-reported risk-taking skiers ski on average 8 km/h faster (measured with a radar speed gun) compared to self-reported cautious skiers [12] and that longer skis with wider turn radii allow for greater ski speed while turning [20]. However, it is also known that when losing control or when a ski error occurs, longer carving skis can act as a longer lever arm to bend or twist the leg leading to a greater risk of an ACL injury [21],[22]. In addition, other ski geometry parameters such as ski widths and standing heights also seem to impact on ACL injury risk during recreational skiing [22]. In fact, Ruedl et al. [22] have recently identified that a higher ski length, a wider ski tip, a higher standing height at the back part of the ski binding and a higher standing height ratio were independent equipment-related risk factors for sustaining an ACL injury. In addition, according to Posch et al. [21] a higher abrasion of the ski boot sole increased ACL injury risk in recreational skiers.

Therefore, to the best of our knowledge, no study has examined whether the choice of ski geometry parameters, especially the width at the tip, waist, and tail of the ski, as well as the standing heights, and amount of ski boot sole abrasion impacts ACL injury risk in cautious as well as in risk-taking recreational skiers. Thus, the aim of this study was to evaluate if and to what extent personal characteristics and equipment-related parameters are associated with ACL injury risk in cautious and risk-taking recreational alpine skiers.

## 2. Materials and methods

### 2.1. Study design

This work was designed as an observational case-control study comparing recreational skiers who self-reported a cautious or risk-taking behaviour on ski slopes. Recreational skiers with and without ACL injuries were evaluated during 4 consecutive winter seasons from 2016/17 to 2019/20 in the same Austrian ski area. The analyzed data was a subset of a recent study across 6 consecutive winter seasons [22] where the observed equipment-related parameters (ski length, side-cut radius, ski widths, standing heights) were assessed from all included study participants.

### 2.2. Subjects

Inclusion criteria were an age > 7 years and the use of any type of carving skis (in contrast to long and unshaped traditional skis). ACL injured skiers (cases) were included when the injury happened after a fall without the involvement of other persons, and subsequently interviewed in a sport clinic situated in this ski area. ACL injuries were diagnosed by using magnetic resonance imaging (MRI) by the consulting physician with expertise in musculoskeletal injury diagnosis.

Uninjured skiers were selected randomly and interviewed by questionnaire at different spots in the ski area throughout the entire ski day, on weekends and during the week.

About 95% of invited subjects agreed to participate in the study. Included skiers gave their written informed consent for participating after being informed about the aims of the study. The survey was conducted according to the ethical guidelines for surveys approved by the Institutional Review Board (IRB) of the Department of Sport Science as well as the Board for Ethical Issues (BfEI) of the University of Innsbruck (certificate of good standing 29/2016).

### 2.3. Questionnaire

Participants were asked for the independent factors of age, sex, body height, and weight. In addition, skiers self-reported their skiing skill level (expert, advanced, intermediate, and beginner) [23] and risk-taking behaviour (risk-taking vs. cautious) [8],[14]. Subsequently, participants were divided into more skilled (expert and advanced) and into less skilled (intermediate and beginner) skiers, as a tendency to underestimate individual skiing skills, especially among female skiers, has been shown in literature [23].

### 2.4. Ski geometry parameters, standing height, and ski boot sole abrasion

Ski length, side-cut radius as well as widths of the tip, waist, and tail of the ski were recorded from the ski of the participant using the manufacturers descriptions printed on the ski.

Standing height (i.e., the distance between the bottom of the running surface of the ski and the ski boot sole) at the front and rear component of the ski binding was measured by the use of a digital sliding caliper and then the percentage ratio between front and rear component of the ski binding (standing high ratio) was calculated according to Ruedl et al. [22].

Ski boot sole abrasion was evaluated by measuring the sole height of the toe and heel piece of the ski boot with the digital sliding calliper and then calculating the difference with the ISO 5355 norm height of ski boot soles (19 ± 1 mm at the toe and 30 ± 1 mm at the heel) as a measure of sole abrasion.

### 2.5. Statistics

Data are presented as means ± standard deviations and absolute or relative frequencies. After checking for normal distribution of metric data with Kolmogorov-Smirnov test, unpaired t-tests or Mann–Whitney-U tests were used to compare uninjured and ACL injured skiers in the cautious group as well as in the risk-taking group with regard to age; body height and weight; ski length; side-cut radius; widths of the tip, waist and tail of the ski; standing height ratio; and sole abrasion of the front and heel piece of the ski boot. In addition, sex, and skiing skill level were compared between risk groups using Chi-square tests. According to previous studies [21], all personal and equipment-related factors with p < 0.1 were additionally entered in a multiple logistic regression analysis (enter method) to estimate adjusted odds ratios (OR) and their 95% confidence intervals (CI) for ACL injury risk in the groups of cautious and risk-taking recreational alpine skiers.

SPSS 26.0 (IBM Corporation, Armonk, NY) was used for the statistical analysis. All p-values were two-tailed and values below 0.05 were considered to indicate statistical significance.

## 3. Results

A total of 1068 recreational skiers (50.8% females) with a mean age of 37.8 ± 12.3 years participated in this study, of whom 193 (22.0%) sustained an ACL injury. The mean body height and weight for the total cohort were 173.1 ± 8.4 cm and 74.5 ± 14.4 kg, respectively. In total, 330 (30.9%) participants reported a “risk-taking behaviour” while skiing.

Moreover, a total of 829 (77.6%) skiers self-reported their skiing ability to be “more skilled”.

Mean absolute ski length, side-cut radius, tip width, waist width and tail width of the total cohort was 165.4 ± 9.8 cm, 14.6 ± 2.9 m, 121.0 ± 8.0 mm, 76.2 ± 9.1 mm, and 106.6 ± 7.9 mm, respectively.

Mean standing height ratio was 88.8 ± 6.9%. Mean ski boot sole abrasion at the toe and heel piece was 2.6 ± 2.4 mm and 3.5 ± 2.8 mm, respectively.

Comparisons of uninjured and ACL injured cautious and risk-taking skiers across all measures are shown in Table 1.

**Table 1.** Univariate comparison of personal and equipment-related risk factors between uninjured and ACL injured cautious and risk-taking skiers.

	**Cautious skiers**			**Risk-taking skiers**		

Risk factors	Uninjured controls (n = 636)	ACL injured cases (n = 102)	P value	Uninjured controls (n = 239)	ACL injured cases (n = 91)	P value
Sex [%]			<0.001			0.004
Male	45.0	20.6		61.6	78.0	
Female	55.0	79.4		38.5	22.0	
Age, mean ± sd	37.5 ± 12.6	42.8 ± 10.0	<0.001	34.0 ± 11.5	44.6 ± 10.0	<0.001
Body height [cm] mean ± sd	172.3 ± 8.4	168.9 ± 6.5	<0.001	175.7 ± 8.8	176.0 ± 6.5	0.967
Body weight [kg] mean ± sd	73.8 ± 14.9	67.7 ± 9.3	<0.001	78.4 ± 15.4	76.2 ± 9.5	0.105
Skill level [%]			<0.001			<0.001
More skilled	78.1	39.2		93.7	64.8	
Less skilled	21.9	60.8		6.3	35.2	
Ski length [cm], mean ± sd	164.3 ± 9.7	161.5 ± 7.4	0.003	169.4 ± 10.6	167.3 ± 6.7	0.050
Sidecut radius [m], mean ± sd	14.4 ± 2.8	13.6 ± 1.9	0.012	15.9 ± 3.4	14.3 ± 2.0	0.001
Tip width [mm], mean ± sd	120.8 ± 7.7	120.3 ± 6.5	0.268	121.7 ± 9.7	120.6 ± 6.5	0.136
Waist width [mm], mean ± sd	75.6 ± 7.9	73.0 ± 4.6	0.001	80.0 ± 12.6	72.9 ± 4.8	<0.001
Tail width [mm], mean ± sd	106.6 ±7.3	104.9 ± 6.9	0.060	108.3 ± 9.5	104.3 ± 7.2	0.001
Standing height ratio [%], mean ± sd	86.9 ± 6.5 (n = 636)	95.3 ± 4.6 (n = 102)	<0.001	88.6 ± 6.2 (n = 239)	95.7 ± 4.5 (n = 91)	<0.001
Ski boot sole abrasion [mm]						
Toe piece, mean ± sd	2.1 ± 2.2	5.0 ± 1.6	<0.001	1.9 ± 2.1	5.4 ± 1.5	<0.001
Heel piece, mean ± sd	3.1 ± 2.8	5.6 ± 1.9	<0.001	2.6 ± 2.7	5.9 ± 1.8	<0.001

Table 2 presents the results of the multiple logistic regression analysis (entering all potential risk factors with p < 0.1 from the simple analysis above) for cautious skiers. A higher age, a lower skill level, a higher ski length, a higher standing height ratio, and greater ski boot sole abrasion at the toe and heel piece are independently associated with an increase in ACL injury risk in cautious skiers.

**Table 2.** Adjusted odds ratios of personal and equipment-related risk factors associated with an ACL injury in cautious skiers.

*Risk factor*	Coefficient	Standard error	*df*	*P*	Odds ratio	95% CI
*Female sex*	0.948	0.625	1	0.129	2.582	0.759–8.783
*Age [years]*	0.043	0.016	1	0.006	1.044	1.012–1.077
*Body height [cm]*	−0.046	0.048	1	0.339	0.955	0.869–1.050
*Body weight [kg]*	−0.018	0.026	1	0.501	0.982	0.933–1.034
*Lower skill level*	1.274	0.384	1	0.001	3.575	1.683–7.592
*Ski length [cm]*	0.099	0.037	1	0.008	1.104	1.026–1.187
*Side-cut radius [m]*	−0.116	0.122	1	0.339	0.890	0.702–1.130
*Waist width [mm]*	−0.055	0.041	1	0.178	0.946	0.873–1.025
*Tail width [mm]*	−0.061	0.036	1	0.092	0.941	0.877–1.010
*Standing height ratio [%]*	0.328	0.042	1	<0.001	1.389	1.278–1.509
*Ski boot sole abrasion at the toe piece [mm]*	0.793	0.128	1	<0.001	2.211	1.719–2.843
*Ski boot sole abrasion at the heel piece [mm]*	0.172	0.080	1	0.031	1.187	1.016–1.388
*Intercept*	−35.599	9.066	1	0.000	0.000	

*Note: Hosmer-Lemeshow-test with p = 0.749, Nagelkerke R-Square = 0.726, classification table – overall percentage = 94.4%.

Results of the multiple logistic regression analysis (entering all potential risk factors with p < 0.1 from the simple analysis above) for risk-taking skiers are shown in Table 3. A higher age, a lower skill level, a higher standing height ratio, and greater ski boot sole abrasion at the toe and heel piece are independently associated with an increase in ACL injury risk in risk-taking skiers.

**Table 3.** Adjusted odds ratios of personal and equipment-related risk factors associated with an ACL injury in risk-taking skiers.

*Risk factor*	Coefficient	Standard error	*df*	*P*	Odds ratio	95% CI
*Female sex*	−0.605	0.754	1	0.423	0.546	0.125–2.396
*Age [years]*	0.097	0.026	1	<0.001	1.102	1.047–1.159
*Lower skill level*	3.145	1.167	1	0.007	23.227	2.356–228.962
*Ski length [cm]*	0.048	0.054	1	0.376	1.049	0.944–1.166
*Side-cut radius [m]*	−0.084	0.165	1	0.610	0.919	0.665–1.271
*Waist width [mm]*	−0.056	0.058	1	0.336	0.946	0.844–1.060
*Tail width [mm]*	0.011	0.047	1	0.813	1.011	0.922–1.109
*Standing height ratio [%]*	0.315	0.060	1	<0.001	1.371	1.219–1.541
*Ski boot sole abrasion at the toe piece [mm]*	0.862	0.202	1	<0.001	2.369	1.596–3.516
*Ski boot sole abrasion at the heel piece [mm]*	0.403	0.129	1	0.002	1.496	1.162–1.926
*Intercept*	−45.476	12.189	1	0.000	0.000	

*Note: Hosmer-Lemeshow-test with p = 0.838, Nagelkerke R-Square = 0.854, classification table – overall percentage = 94.8%.

## 4. Discussion

This study evaluated if personal and equipment-related factors are associated with ACL injury risk in skiers with a cautious and with a risk-taking behaviour. Results of the multiple analysis found that a higher age, a lower skill level, a higher standing height ratio, and greater ski boot sole abrasion at the toe and heel piece were independently associated with an increased ACL injury risk irrespective of risk-taking behaviours. In cautious skiers, a longer ski length was an additional risk factor for sustaining an ACL injury.

### 4.1. Age and skill level

Mean age of cautious and risk-taking skiers was 38.2 and 36.9 years, respectively. There is evidence that risk-taking behaviour on ski slopes is associated with lower age [12],[16],[17],[19] and that in general risk-taking behaviour might be associated with the personality trait sensation seeking [15] with a significant age decline in sensation seeking scores in both sexes, especially in the Thrill and Adventure Seeking scale [24]. However, ACL injury risk significantly increased with increasing age in cautious as well as in risk-taking skiers. In the cautious cohort ACL injured skiers were on average 5 years older (38 vs. 43 years) and in the risk-taking cohort on average 11 years older (34 vs. 45 years). In recreational skiing, higher age as a risk factor for knee/ACL injuries is well known [22],[25]. These age-related factors include a progressive loss of neuromuscular function accompanied by a reduction of muscle quantity and quality in conjunction with changes in the biology, healing capacity, and biomechanical function of tendons and ligaments [26],[27]. How these age-related effects in knee function affected ACL injury occurrence is unclear in the present study; however, our data does illustrate that regardless of ski behaviour older individuals make up the ACL injury groups.

Furthermore, the proportion of more skilled skiers was higher in the risk-taking compared to the cautious group (88.5 vs. 72.8) which is similar to existing literature [12],[16],[17],[19]. However, independent of risk behaviours, a lower skill level also significantly increased ACL injury risk 4-fold in cautious and by 23-fold in risk-taking skiers, respectively. It is well known from research that the lower the skill level of winter sport participants the higher the overall injury risk on alpine ski slopes [28],[29]; the present study confirms these findings. In particular, regarding ACL injuries, it is also known that lower skill level is an independent risk factor for sustaining an ACL injury [19],[21],[22]; however, our results indicate that being a risk-taking skier with low skill level alters the risk of a knee injury. This finding illustrates the combinative effect that risk behaviour and skill level has on injury risk and that skiing slow and controlled is very protective in less skilled skiers against their knee injury risk. However, this result should be interpreted with caution given the large confidence interval associated with these increased odds of injury in less skilled skiers. The reason for the large confidence interval is not completely clear, thus the more conservative conclusion from this finding would be that odds of injury are increased in less skilled risky skiers, but the magnitude of the odds is not conclusive.

### 4.2. Ski length and standing height ratio

Results of the multiple logistic regression analysis revealed that a greater mean ski length (adjusted for body height) was significantly associated with an increase in ACL injury risk in cautious skiers only. Generally, longer skis have been demonstrated to increase the ACL injury risk [21],[22]. This is not surprising as, e.g., in the case of a fall, longer skis act as a longer lever arm, elevating the torques at the knee joint and the associated injury risk [22]. Why only cautious skiers are affected remains to be elucidated, but may be associated with the mode of skiing and/or falling.

Mean difference of the standing height ratio (i.e., the percentage ratio between front and rear component of the ski binding) was significantly higher in ACL injured skiers of the cautious (95 vs. 87%) as well as of the risk-taking group (96% vs. 89%) compared to uninjured skiers. In comparison, Ruedl et al. [22] who has introduced this novel parameter recently, found that ACL injured skiers, regardless of sensation seeking intentions, did have a mean standing height ratio of approximately 95% (a more horizontal ski boot) compared to approximately 89% (similar to plantarflexion rotation of the ski boot) in uninjured skiers. Given that ski boot orientation can influence knee kinematics and would likely alter the loads experienced at the knee [30], it was speculated that a higher heel position of the ski boot potentially shifts the skier's center of gravity forward allowing a better steering of the skis [22]. In addition, an elevated heel position of the ski boot could cause a more forward trunk lean position of the skier and likely increase knee flexion (moving the knee further from an extended position) which is associated with a decreasing load on the ACL [31]. Our results would also indicate that the standing height ratio seems to be a relevant factor associated with ACL injury independent of individuals' risk behaviours. Thus, cautious and risk-taking skiers should be aware of the potential preventive impact of a lower standing height ratio on ACL injury risk [22].

### 4.3. Ski boot sole abrasion

Abrasion of the toe and heel piece of the ski boot sole significantly increased ACL injury risk by 1.2-2.4-fold in the cautious as well as in the risk-taking cohort. In accordance, a study by Posch et al. [21] has found that the abrasion at the toe and the heel of the ski boot may increase such risk 1.4 to 1.8 times, however, without evaluating potential differences based on skiers' risk-taking behaviours. Causes for a significant abrasion of a ski boot sole could be an excessive walking on ski boots on surfaces different from snow, e.g., the streets or parking areas of ski resorts [21]. One could speculate that abrasion of the ski boot sole can negatively influence the release mechanisms of the ski binding because the ski boot sole acts as the key interface between forces produced by the skier and the ski binding [32]. Thus, any abrasion which might affect the ability to release the boot from the ski likely has a real-world effect on forces that occur when a skier is falling or catching an edge. Regardless of how sole abrasion has occurred, it is clear that protecting boot sole is going to reduce the probability of an ACL injury, and that in risk-taking skiers, protecting soles is even more important (likely due to the rate of speed and forces induced in turning at higher speeds). However, more studies are needed to examine the abovementioned role of ski boot sole abrasion with regard to unexpected release or failure of release of a ski binding, and is a novel avenue of future research with ski boot manufacturers.

### 4.4. Limitations

A few limitations have to be considered. Firstly, we only recruited injured skiers from the key clinic in the ski area. It is unlikely that individuals who sustained an ACL injury at the ski area would have bypassed the clinic [33]; however, this possibility does exist and may have had an influence on the total number of ACL injuries recorded. Second, selection bias in the recruitment of the control group cannot be excluded. However, as you can't predict when and where skiers suffer an injury, the random selection of uninjured skiers at different spots in the ski area of interest, as already shown in the literature [21], has probably been the only practical method to include a control group. Third, because a single question was used to assess risk-taking behaviour, this might lead to under-reporting or over-reporting of risk behaviours determination; indeed, cognitive and situational factors, especially in younger people, can affect the response to a question [34]. However, this question to assess risk-taking behaviour (i.e., risk-taking vs. cautious) on ski slopes appears to have face validity [8] and seems to be a valid single item approach related to the sensation seeking total score as self-reported risk-taking behaviour was significantly associated with a higher sensation seeking total score compared to the reported cautious behaviour of skiers and snowboarders (sensation seeking score in risk-takers = 23.8 vs. 20.3 in cautious assessed with the German version of the sensation seeking scale form V) [13]. Furthermore, Jack & Ronan [35] reported a mean sensation seeking total score (also assessed with the sensation seeking scale form V) of 23.0 among high-risk sports participants (hang-gliders, mountaineers, sky-divers, automobile racers) and of 20.3 among low-risk sports participants (golfers, swimmers, marathon runners, aerobics), whereby the link between sensation seeking and risk-taking is well grounded [36].

## 5. Conclusions

In conclusion, personal characteristics such as higher age and lower skill level as well as specific equipment related characteristics (higher values of the standing height ratio and ski boot sole abrasion) are associated with an increase in the ACL injury risk in cautious as well as risk-taking skiers, with the only difference that longer skis represent an additional risk factor in cautious skiers. These findings may help provide recommendations to skiers concerning the selection of appropriate equipment in order to reduce the ACL injury risk. An important aspect of this would be to work with ski areas and the snow sports equipment industry to provide some basic highlights that show how equipment might influence the skiers' safety and risk of injury.
